# Natural hybrid of *Leishmania infantum*/*L. donovani*: development in *Phlebotomus tobbi*, *P. perniciosus* and *Lutzomyia longipalpis* and comparison with non-hybrid strains differing in tissue tropism

**DOI:** 10.1186/s13071-015-1217-3

**Published:** 2015-11-25

**Authors:** Veronika Seblova, Jitka Myskova, Jana Hlavacova, Jan Votypka, Maria Antoniou, Petr Volf

**Affiliations:** Department of Parasitology, Charles University, Faculty of Science, Prague 2, Czech Republic; Laboratory of Clinical Bacteriology, Parasitology, Zoonoses and Geographical Medicine, Faculty of Medicine, University of Crete, Crete, Greece

**Keywords:** *Phlebotomus tobbi*, Vector competence, *Leishmania* development, Tropism

## Abstract

**Background:**

Infection caused by parasites from *L. donovani* complex can manifest as a serious visceral disease or a self-healing milder cutaneous form. The different tropism and pathology in humans is caused by the interaction between parasites, host and vector determinants but the mechanisms are not well understood. In Cukurova region in Turkey we previously identified a major focus of cutaneous leishmaniasis caused by *L. donovani/infantum* hybrids (CUK strain) and isolated this parasite from the locally abundant sand fly, *Phlebotomus tobbi*. Here, we present the first experimental study with *P. tobbi*. We tested the susceptibility of this species to various *Leishmania* under laboratory conditions, characterized glycoproteins in the *P. tobbi* midgut putatively involved in parasite-vector interaction and compared the development of the CUK strain in the sand fly with one other dermotropic and three viscerotropic strains belonging to the *L. donovani* complex.

**Methods:**

Females of laboratory reared *P. tobbi*, *P. perniciosus* and *Lutzomyia longipalpis* were infected using membrane feeding on rabbit blood containing promastigotes of various *Leishmania* species with different tropisms. The individual guts were checked microscopically for presence and localization of *Leishmania* parasites; the number of parasites was assessed more precisely by qPCR. In addition, glycosylation of midgut proteins of *P. tobbi* was studied by lectin blotting of midgut lysate with lectins specific for terminal sugars of N-type and O-type glycans.

**Results:**

High infection rates, heavy parasite loads and late-stage infection with colonization of the stomodeal valve were observed in *P. tobbi* infected by *Leishmania major* or *L. infantum* CUK hybrid. In parallel, lectin blotting revealed the presence of O-glycosylated proteins in the *P. tobbi* midgut. In *P. perniciosus* and *L. longipalpis* all five *Leishmania* strains tested developed well. In both vectors, significantly higher parasite numbers were detected by qPCR for dermotropic *L. donovani* from Cyprus, however, in all other parameters studied, including localization of infection and colonization of stomodeal valve, dermotropic and viscerotropic strains were not significantly different.

**Conclusions:**

We showed high susceptibility of *P. tobbi* to various *Leishmania* spp. This, together with the presence of O-glycosylated midgut proteins in their midguts demonstrate that *P. tobbi* is a permissive vector. Two dermotropic and three viscerotropic strains from the *L. donovani* complex developed late-stage infections in natural *L. infantum* vectors, *P. perniciosus* and *L. longipalpis* and none of the parameters studied seem to be linked with different tropism of parasites in the vertebrate host.

## Background

The *Leishmania donovani* complex comprises two generally recognized species, *L. infantum* (syn. *chagasi*) and *L. donovani*. These parasites usually cause a serious visceral disease, however, both *L. infantum* and *L. donovani* can also manifest as a milder cutaneous form of leishmaniasis. In such cases, lesions occuring in patients are relatively small, non-ulcerating and have a tendency to self-heal. Cutaneous leishmaniases caused by *L. infantum* and *L. donovani* have been reported worldwide, including countries of the Mediterranean basin, and recently were found to be expanding northward to other European countries [1].

In the Old World, parasites belonging to *L. donovani* complex are transmitted by more than twenty-five different species of the genus *Phlebotomus*, while in the New World *Lutzomyia longipalpis* serves as the main vector [2]. Most of these sand fly species support development of multiple *Leishmania* species. They are called permissive vectors [3, 4] and are characterized by the presence of O-glycosylated proteins with N-acetylgalactosamine (GalNAc) epitopes on the microvillar surface of the midgut, specifically recognized by lectin *Helix pomatia* agglutinin (HPA) [5].

A major focus of cutaneous leishmaniasis caused by parasites of the *L. donovani* complex was described in Cukurova region in Turkey [6]. Multilocus enzyme electrophoresis showed that these Cukurova (CUK) strains belong to a new zymodeme MON-309 and are genetically closely related to Cypriot strains previously typed as *L. donovani* zymodeme MON-37 [7]. Recently, whole genome sequencing demonstrated that CUK strains are the progeny of a single outcrossing event between two diverse parents, one likely to be an *L. donovani* and the second similar to *L. infantum* JPCM5 [8].

Field results strongly suggest *Phlebotomus tobbi* as a vector of CUK strains: all sand fly females found to be *Leishmania*-positive were identified as *P. tobbi* [6]. This sand fly species is widely distributed in the Eastern part of the Mediterranean and Middle East and belongs to the *Larroussius* subgenus comprising several proven vectors of visceral leishmaniases [9]. Previously, natural infection of *P. tobbi* with *L. donovani* or *L. infantum* were reported from Syria and Cyprus, respectively [10–12], and *Leishmania infantum* DNA was repeatedly found in *P. tobbi* in northwestern Iran [13, 14]. Nevertheless, detailed information about the vector competence of *P. tobbi* is absent. Here, we established a *P. tobbi* colony and studied, for the first time, the development of CUK hybrids in their natural vector.

Additionaly, we tested a hypothesis that the tropism and severity of the *L. infantum* and *L. donovani* infection outcome in the host is caused by different development patterns of dermotropic and viscerotropic strains in sand flies. In *Leishmania major*, the severity of infection outcome is supposed to be affected by variation in numbers of inoculated promastigotes into the host skin by individual female sand flies during feeding. There is a great deal of variability in parasite transmission by a single bite from less than 10 up to 100 000 promastigotes and this might be an inherent character of each *Leishmania* strain and sand fly species involved [15–18]. Hypothetically, the high number of transmitted parasites could promote a strong local immune response in the dermis and eliminate dissemination of parasites to visceral organs so that only cutaneous disease manifests [17]. We tested the development of CUK hybrids in *P. tobbi* and in two natural vectors of *L. infantum*, the Old World *P. perniciosus* and the New World *L. longipalpis,* and compared it with one dermotropic and three viscerotropic strains from the *L. donovani* complex.

## Methods

### Parasites

*Leishmania major* LV561 (MHOM/IL/67/LRC-L137 Jericho-II) and five strains of parasites from the *L. donovani* complex were used: dermotropic *L. infatum/donovani* hybrid from Cukurova, Turkey (CUK3: ITOB/TR/2005/CUK3) [7], dermotropic *L. donovani* from Cyprus (CYPR: MHOM/CY/2011/592/11) [19], viscerotropic *L. infantum* from Turkey (OG-VL: MHOM/TR/2000/OG-VL), viscerotropic *L. infantum* from Portugal (IMT373: MCAN/PT/2005/IMT373) [20] and viscerotropic *L. donovani* from Ethiopia (GEBRE: MHOM/ET/72/GEBRE1) [7], M.A. unpublished data. All parasites were maintained at 23 °C in M199 medium supplemented with 10 % foetal calf serum (Gibco), 1 % BME vitamins (Sigma), 2 % sterile urine and 250 μg/ml amikin (Amikin, Bristol-Myers Squibb). Before experimental feeding, parasites were washed by centrifugation and resuspended in saline solution.

### Sand fly colonies

The colony of *P. tobbi* was established in 2005 from specimens collected in the leishmaniases focus in Cukurova region, Adana province, southern Turkey. Detailed information about the colonization and rearing of sand flies, including *P. tobbi,* are summarized in Volf and Volfova [21]. The colony appears to be very difficult to maintain; adult females require human or rabbit bloodmeal and have a high post-bloodmeal mortality. In contrast to most other sand fly species maintained in our facilities, *P. tobbi* females refused to feed on mice and were reluctant to feed through any type of artificial membrane. After seven years of struggling with the establishment of a *P. tobbi* colony we achieved sufficient numbers of females to study their susceptibility to *L. major* and the CUK hybrid isolate.

Well-established laboratory colonies of proven vectors of *L. infantum*, *Phlebotomus perniciosus* (originating from Spain) and *Lutzomyia longipalpis* (Brazil-Jacobina), were used for comparison of development of various strains from the *L. donovani* complex. All experiments were done at 26 °C, on a 14-h light/10 dark photoperiod and sand flies had free access to 50 % sucrose solution.

### Experimental infection of sand flies

Sand fly females were infected by feeding through a chick-skin membrane on suspension of promastigotes mixed 1:10 with heat-inactivated rabbit blood (Bioveta, Ivanovice na Hane). The final concentration of parasites was 1 × 10^6^ promastigotes/ml. Engorged females were separated and maintained in the conditions described above. On days 2 and 7–9 post-bloodmeal (PBM) females were dissected in a drop of saline and examinated by light microscopy. Intensity of infection were graded to three categories as weak (<500 parasites/gut), moderate (500–1000 parasites/gut and heavy (>1000 parasites/gut) as described previously [22]. More precise quantification of the numbers of *Leishmania* parasites in the guts of female sand flies was performed by qPCR using the SYBR Green detection method (Biorad) target on amplification of *Leishmania* kinetoplast DNA under conditions described in Mary et al. [23]. Data were evaluated by the Kruskal-Wallis test (STATISTICA 6.1, StatSoft).

### Detection of glycoproteins in sand fly midgut

Glycosylation of midgut proteins of *P. tobbi* was studied by affinity blotting of midgut lysate with lectins specific for terminal sugars of N-type and O-type glycans. Sand fly midguts were dissected from 3–10 day-old females. Midgut proteins were analysed by SDS PAGE on 10 % gel under reducing conditions (15 female midgut per lane) followed by Western blotting. The nitrocellulose membrane was blocked by overnight incubation at 4 °C in 20 mM Tris, 150 mM NaCl, 0,05 % Tween, pH 7,6 (TBS-Tw) with 5 % bovine serum albumin. Then, the membrane was incubated for 1 h with biotinylated lectins *Helix pomatia* agglutinin (HPA), 3 μg/ml and concanavalin A (Con A), 0.5 μg/ml. In the control groups, lectins were preincubated with carbohydrate inhibitor for 30 min. We used 250 mM N-Acetyl-galactosamine (GalNAc) for HPA and 250 mM Methyl α-D-mannopyranoside (MetMan) for Con A. After repeated washing in TBS-Tw blots were incubated with streptavidin peroxidase (2.5 μg/ml) in TBS-Tw for 1 h. The peroxidase reaction was developed in 4-chloro-1-naphtol solution. All chemicals were purchased from Sigma.

### Ethics statement

Animals were maintained and handled in the animal facility of Charles University in Prague in accordance with institutional guidelines and Czech legislation (Act Number 246/1992 and 359/2012 coll. on Protection of Animals against Cruelty in present statutes at large), which complies with all relevant European Union and international guidelines for experimental animals. The experiments (including sand fly feeding) were approved by the Committee on the Ethics of Animal Experiments of the Charles University in Prague (Permit Number 24/773/08-10001) and were performed under the Certificate of Competency (Registration Number CZU945/05 ext. CZ02573) and the Permission Number 31114/2013-MSMT-13 ext. 24115/2014-MZE-17214 of the Ministry of the Environment of the Czech Republic.

## Results

### Susceptibility of *P. tobbi *to *Leishmania*

**Fig. 1:**
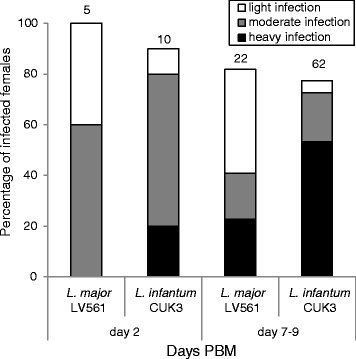
Development of *L. major* (LV561) and *L. infantum* (CUK3) in *Phlebotomus tobbi.* Rates and intensities of infections in *P. tobbi* females examined 2 and 7–9 days PBM (post-bloodmeal) using light microscopy. The infection intensities were classified into three categories according to their intensity: heavy (more than 1000 parasites per gut [black]); moderate (500–1000 parasites [grey]) and light (less than 500 parasites [white]). Numbers above the bars indicate the total number of dissected sand fly females. Data are summarised from eight independent experiments with *L. major* (LV561) and fourteen experiments with *L. infantum/donovani* (CUK3)

*Phlebotomus tobbi* is difficult to maintain under laboratory conditions and extremely difficult to perform experimental infections with (for details see Methods). Therefore, the number of *Leishmania* strains tested was limited to *L. major* LV561 and *L. infantum/donovani* CUK3 hybrid. As only a few *P. tobbi* females took an infective bloodmeal in each experimental feeding, the experiment was repeated eight times with *L. major* and fourteen times with CUK3 strain. Results are summarized in Fig. 1.

In the early stage of infection (2 days PBM) the infection rates of *P. tobbi* females were between 90–100 % for both *Leishmania* species tested. Parasites multiplied inside the bloodmeal bolus surrounded by peritrophic matrix, with more intensive infections found in females infected with *L. infantum* (CUK3). In the late stage infections (7–9 days PBM), the infection rate of both *Leishmania* species were similar (around 75 %) (Fig. 1). However, the intensities of infection were significantly higher in *P. tobbi* infected with CUK3; *L. infantum/donovani* hybrids established heavy infections, migrated anteriorly to the thoracic midgut and colonized the stomodeal valve in 80 % of infected females. In contrast, intensities of infection by *L. major* LV561 were lower; heavy or moderate late-stage infections were observed in 40 % of females and the stomodeal valve was colonized in 11 % of infected *P. tobbi* females.

### Glycosylation of *P. tobbi* midgut proteins

Susceptibility of *P. tobbi* to both parasite strains tested suggests that this sand fly species is a permissive vector in the sense of Volf and Myskova [5]. All permissive sand fly vectors studied up to date are characterized by O-glycosylated epitopes in the midgut epithelium [24]. We analysed glycosylation of midgut proteins of *P. tobbi* using Western blot with lectins specific for sugars which are typical components of N- and O-glycans. Midguts of *L. longipalpis* and *P. papatasi*, sand flies with known midgut glycosylation [4], were used as positive and negative controls, respectively.

**Fig. 2:**
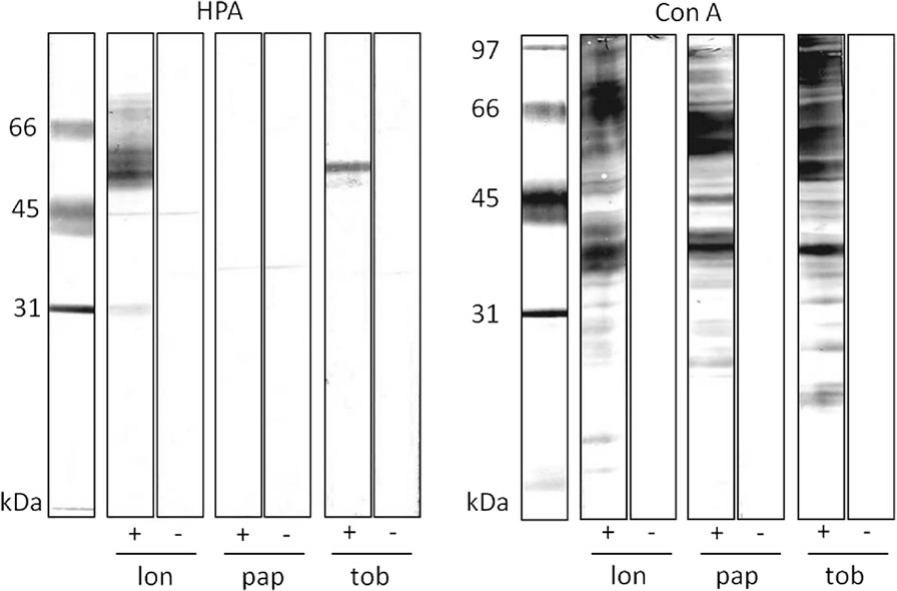
Sand fly midgut lysates separated by SDS PAGE, blotted and incubated with biotinylated lectins. SDS PAGE and blotting of three midgut lysates from *Lutzomyia longipalpis* (lon), *Phlebotomus papatasi* (pap) and *P. tobbi* (tob) with lectins: concanavalin A (Con A) and *Helix pomatia* agglutinin (HPA). + reaction with lectin; - preincubation of the lectin with specific sugar (GalNAc for HPA and MetMan for Con A); HPA specifically reacted with *L. longipalpis* and *P. tobbi*

All midgut lysates studied displayed glycoproteins that bind concanavalin A. This lectin is specific for terminal mannose residues present in N-linked glycans. On the other hand, reaction of lectin HPA, specific for N-acetylgalactosamine and indicating O-type of glycosylation [25], was detected only in *P. tobbi* and in *L. longipalpis* (used as a positive control). Specificity of HPA binding was confirmed by the negative reaction on strips where lectin was preincubated with its inhibitory carbohydrate GalNAc (Fig. 2).

### Development of dermotropic and viscerotropic parasites in sand fly vectors

Experimental infections with the CUK3 hybrid and four other *Leishmania* strains differring in tropism were studied in two natural vectors of visceral leishmaniasis, *L. longipalpis* and *P. perniciosus.*

**Fig. 3:**
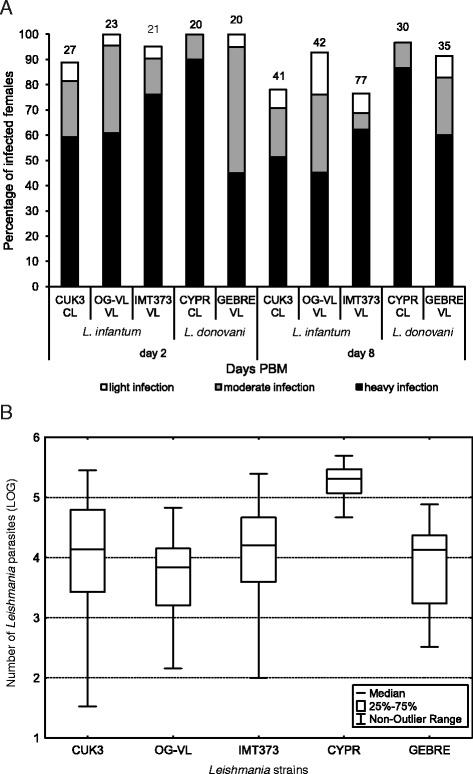
Development of five strains from *Leishmania donovani* complex in *Lutzomyia longipalpis.* **a**: Infection rates and intensities (weak, moderate, heavy) of five strains from *L. donovani* complex: CUK3 (dermotropic *L. infantum*), OG-VL (viscerotropic *L. infantum*), IMT373 (viscerotropic *L. infantum*), CYPR (dermotropic *L. donovani*) and GEBRE (viscerotropic *L. donovani*) in *L. longipalpis* determined on days 2 and 8 PBM (post-bloodmeal) using light microscopy. The infection intensities were classified into three categories according to their intensity: heavy (more than 1000 parasites per gut [black]); moderate (500–1000 parasites [grey]) and light (less than 500 parasites [white]). Numbers above the bars indicate the total number of dissected sand flies. Data are summarised from at least two independent experiments. **b**: Analysis of the parasite loads by quantitative PCR (qPCR) in *L. longipalpis* on day 8 PBM. Thirty to sixty midguts were analysed for each line

**Fig. 4:**
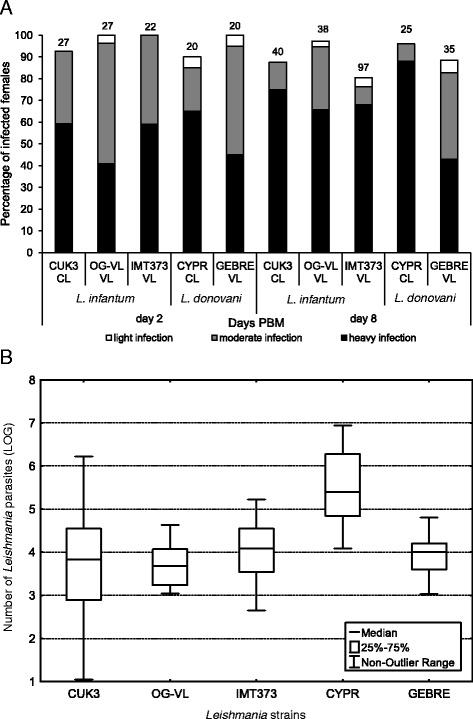
Development of five strains from *Leishmania donovani* complex in *Phlebotomus perniciosus.* **a**: Infection rates and intensities (weak, moderate, heavy) of five strains from *L. donovani* complex: CUK3 (dermotropic *L. infantum*), OG-VL (viscerotropic *L. infantum*), IMT373 (viscerotropic *L. infantum*), CYPR (dermotropic *L. donovani*) and GEBRE (viscerotropic *L. donovani*) in *P. pernicicosus* examined on days 2 and 8 PBM (post blood meal) using light microscopy. The infection intensities were classified into three categories according to their intensity: heavy (more than 1000 parasites per gut [black]); moderate (500–1000 parasites [grey]) and light (less than 500 parasites [white]). Numbers above the bars indicate the total number of dissected sand fly females. Data are summarised from at least two independent experiments. **b**: Analysis of the parasite loads by quantitative PCR (qPCR) in *P. pernicicosus* on day 8 PBM. Thirty to sixty midguts were analysed for each line

On day 2 PBM, microscopical examination of dissected midguts revealed that all studied *Leishmania* strains produced moderate or heavy infection in more than 75 % of sand fly females; the highest infection rates (100 %) were observed for two *Leishmania* strains, CYPR in *L. longipalpis* and IMT373 in *P. perniciosus* (Fig. 3a and Fig. 4a). Nevertheless, statistical analysis showed that localization of early-stage infections and midgut infection rates did not significantly differ between all parasite-vector combinations (*P* > 0.05).

On day 8 PBM, all studied parasite strains continue to develop successfully in *L. longipalpis* and *P. pernicicosus*. In all parasite-vector combinations the infection rates were above 75 % and parasites developed heavy late-stage infections (Fig. 3a and Fig. 4a). Microscopical examination did not show any significant differences in localization of parasites in the thoracic or abdominal midgut between all *Leishmania* strains (*P* > 0.05), slight differences were found only in the colonization of the stomodeal valve. In both sand fly species tested, the colonization of the stomodeal valve was observed most frequently for OG-VL and CYPR (90–100 % of infected females), followed by CUK3 and GEBRE (80–85 %) and IMT373 (65 % of infected females).

To estimate precise numbers of parasites *in situ*, microscopy counts were supplemented with qPCR from 30–50 females of each *Leishmania* strain-vector combination. On day 8 PBM the *Leishmania* numbers were similar for CUK3, OG-VL, IMT 373 and GEBRE strains, however, significantly higher parasite numbers were detected in *L. longipalpis* and *P. perniciosus* females infected by CYPR (*P* < 0.001) (Fig. 3b and Fig. 4b).

### Discussion

The incrimination of a sand fly species as a vector is based on a series of criteria summarized by Killick-Kendrick [24] that are still widely accepted [2]. These criteria include: 1) anthropophily of suspected sand fly species and, if zoonotic transmission is considered, 2) willingness to feed on the reservoir hosts. Then, 3) parasites isolated from the sand fly must be identical to those found in human patients, 4) parasites produce the late-stage infection (including colonization of stomodeal valve) in suspected sand fly species. Additionally, 5) the vector should be able to transmit the parasite by the bite [24]. Evidence that *P. tobbi* meets the first three criteria for parasites of the *L. donovani* complex originates from the field work in Cukurova region [6]. Here, we demonstrated complete development of a CUK hybrid isolate in *P. tobbi* after the infecting bloodmeal had been digested, fulfilling the fourth criterion for vector incrimination.

The results with CUK strain flourishing in *P. tobbi* are in accordance to field surveys conducted in Cyprus [11] and Syria [10] where isolated promastigotes from *P. tobbi* were characterized as *L. infantum* and *L. donovani*, respectively. Recent studies reported this sand fly species positive for presence of *L. infantum* DNA in northwestern Iran [13, 14] and northern Cyprus [12]. The permissivity of *P. tobbi* together with its oportunistic feeding habit [11, 27] and high attraction to humans [6] make *P. tobbi* an important vector of cutaneous and visceral leishmaniasis throughout the eastern part of Mediterranean basin and Middle East.

Our experiments revealed high susceptibility of *P. tobbi* to various *Leishmania* species, namely *L. major* and *L. infantum* CUK3 hybrids. Both parasites tested survived bloodmeal digestion well, avoided the expulsion during the defaecation process, established late stage infections in *P. tobbi* midgut and colonized the stomodeal valve of the sand fly. This, together with the presence of O-glycosylated midgut proteins with GalNAc epitopes, allow us to identify *P. tobbi* as a permissive vector [4]. Leishmaniasis is a multifactorial disease and it is not known which aspects are decisive in outcome and the consequent manifestation of infection with parasites from the *L. donovani* complex as cutaneous or visceral disease [26]. The key role in parasite virulence play genetic differences between *L. infantum* and *L. donovani* subspecies [27] and high levels of *L. infantum* zymodemes heterogenity as well as host immune status [28] and genetic background with marked variation within gene polymorphism in diverse human population (reviewed in [29, 30]. Specifically, HIV-*Leishmania* co-infected patients show a high prevalence of visceralising infections with usually dermotropic *L. infantum* strains [31] including unusual secondary localization of parasites in their body [32]. Here, however, we studied CUK3 hybrids causing exclusively cutaneous disease in an immunocompetent population within a focus of over 50 km [6, 33]. We tested the hypothesis that the tropism of the parasite and the manifestation of the disease might be affected by different development patterns in the sand fly vector. Development of CUK3 hybrids in two sand fly species was compared with one dermotropic and three viscerotropic strains, all belonging to the *L. donovani* complex. Dermotropic *L. donovani* from Cyprus (CYPR) produced significantly heavier infections than other strains in both vectors, however, in other parameters, like the percentage of stomodeal valve colonization, all strains were comparable. The growth rate observed in the culture was similar to the other four strains studied and did not explain why dermotropic *L. donovani* from Cyprus produced significantly heavier infections. Sand fly infections by dermotropic CUK3 hybrids were similar in all parameters to the three viscerotropic strains. In conclusion, our results revealed that dermotropic parasites from *L. donovani* complex are very infective for sand flies and produce late stage infections similar to viscerotropic strains.

The infectivity of dermotropic and viscerotropic *L. infantum* strains have been investigated using *in vitro* experiments on monocyte-macrophage cultures infected by *Leishmania* promastigotes and using a mouse model. The *in vitro* approach did not provide any evidence that tropism of *L. infantum* strains in humans is associated with different infectivity for macrophages [34–36] and *in vivo* studies led to contradictory results: while Sulahian et al*.* [37] reported a CL strain as rather avirulent and never visceralizing in mice, Cunha et al*.* [38] showed a single dermotropic strain to be more infective (able to produce high parasite numbers also in internal organs) than 3 viscerotropic strains. However, the reliability of these in-vivo findings is diminished by the route of inoculation adopted (intraperitoneal and intravenous), which does not reflect the complexity of transmission by a sand fly bite. Particularly, addition of saliva and promastigote secretory gel to inoculum, which are very effective virulence immunomodulatory factors, lead to immunomodulation in the site of bite/inoculation and more serious manifestations both in the cutaneous and the visceral forms of leishmaniasis [39, 40].

## Conclusions

We demonstrated a successful development of CUK3 hybrids in their natural sand fly vector *P. tobbi* and in two *L. infantum* vectors, *P. perniciosus* and *L. longipalpis*. Comparison with another four leishmania strains from the *L. donovani* complex revealed slight differences in parasite loads during late-stage infections. However, none of these parameters seem to be linked to tropism of the parasites and infection outcome in the vertebrate host. In conclusion, dermotropic strains developed in vectors comparably to the viscerotropic ones.

## Abbreviations

GalNAc: N-acetylgalactosamine
PBM: post-bloodmeal
ConA: Concanavalin A
HPA: *Helix pomatia* agglutinin
MetMan: Methyl α-D-mannopyranoside
TBS-Tw: Tris-buffered saline-Tween
